# A de novo *MAPRE2* variant in a patient with congenital symmetric circumferential skin creases type 2

**DOI:** 10.1002/mgg3.1096

**Published:** 2020-01-05

**Authors:** Jincai Feng, Xiaoping Lan, Jun Shen, Xiaozhen Song, Xiaojun Tang, Wuhen Xu, Xiang Ren, Hong Zhang, Guangjun Yu, Shengnan Wu

**Affiliations:** ^1^ Department of Shanghai Children's Hospital Shanghai Jiaotong University Shanghai China; ^2^ Department of Pathology Brigham and Women's Hospital Harvard Medical School Boston MA USA

**Keywords:** congenital symmetric circumferential skin creases, exome sequencing, *MAPRE2*, mutations

## Abstract

**Background:**

Congenital symmetric circumferential skin creases (CSCSC) was initially described five decades ago. Exome sequencing has recently revealed the genetic etiology of CSCSC. Pathogenic variants in *TUBB* (OMIM# 191130) and *MAPRE2* (OMIM# 605789) have been linked to CSCSC1 (OMIM# 156610) and CSCSC2 (OMIM# 616734), respectively, in an autosomal dominant manner. Four pathogenic variants in *MAPRE2* have been previously reported to be associated with CSCSC2.

**Methods:**

Whole‐exome sequencing (WES) has been performed and an in‐house pipeline was used to conduct a phenotype‐driven data analysis. All candidate variants were confirmed by Sanger sequencing.

**Results:**

Here we report a 2‐year‐old boy characterized by absent expressive speech, normal to mild over growth, facial dysmorphic features, remarkable circumferential skin creases on both forearms and ankles. WES disclosed a de novo missense MAPRE2 variant, c.518G>A (p.Arg173Gln), as the molecular cause of this complex phenotype. We described detailed clinical characterization of this patient and compared the available clinical data of individuals with MAPRE2 variants to demonstrate the phenotypic spectrum.

**Conclusion:**

Our study reports the first patient of Asian origin with CSCSC2 due to a pathogenic mutation of MAPRE2 and expands the clinical and genetic spectrum of CSCSC2.

## Graphical abstract

A de novo MAPRE2 variant has been detected using whole exome sequencing in a patient with congenital symmetric circumferential skin creases, who has different clinical phenotypes compared with previously reported patients. This findings have expanded the clinical spectra of MAPRE2‐related disorder.

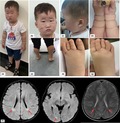

## 1 INTRODUCTION

Congenital symmetric circumferential skin creases (CSCSC) was initially described as “Michelin tire baby”, and subsequent reports described it as “Michelin tire baby syndrome” with additional clinical features, which was typically characterized by the ringed creases of limbs and neck accompanied by intellectual disability, speech delay, short stature, facial dysmorphism, microcephaly, and various abnormality of fingers and toes (Ross, 1969). CSCSC includes two different types, CSCSC1 (OMIM# 156610) and CSCSC2 (OMIM# 605789), due to different molecular bases. The clinical information of this disorder had already been widely delineated before the underlying genetic basis was discovered (Basel‐Vanagaite et al., 2012; Cohen, Gorlin, Clark, Ewing, & Camfield, 1993; Elliott, Ludman, & Teebi, 1996; Leonard, 2002; Ulucan et al., 2013; Wouters et al., 2011). A limited published cohort study has established a few genotype–phenotype correlations of two genes and CSCSC (Isrie et al., 2015), reporting three missense and one nonsense variants in *MAPRE2* within the calponin–homology domain and three missense variants in *TUBB*. *MAPRE2* encodes a microtubule‐associated protein, which is a central regulator of microtubule dynamics and reorganization during cell differentiation (Goldspink et al., 2013). *TUBB* is one of the nine human beta‐tubulin genes, which play a crucial role in the mechanisms of central nervous system development (Romaniello, Arrigoni, Bassi, & Borgatti, 2015). Another *TUBB* variant has recently been reported in a boy with a diagnosis of CSCSC1 (Dentici et al., 2018). However, no additional *MAPRE2* variants have been detected except for the initial findings.

Here we report a 2‐year‐old male characterized by absent expressive speech, normal to mild over growth, facial dysmorphic features, remarkable circumferential skin creases on both forearms and ankles. Exome sequencing on the proband and both parents identified a novel de novo pathogenic missense variant in *MAPRE2* in the calponin–homology domain. Our findings diversify the clinical features of this rare disease in Chinese Han ethnicity and strengthen the association of *MAPRE2* with CSCSC.

## 2 CLINICAL DESCRIPTION

**Figure 1:**
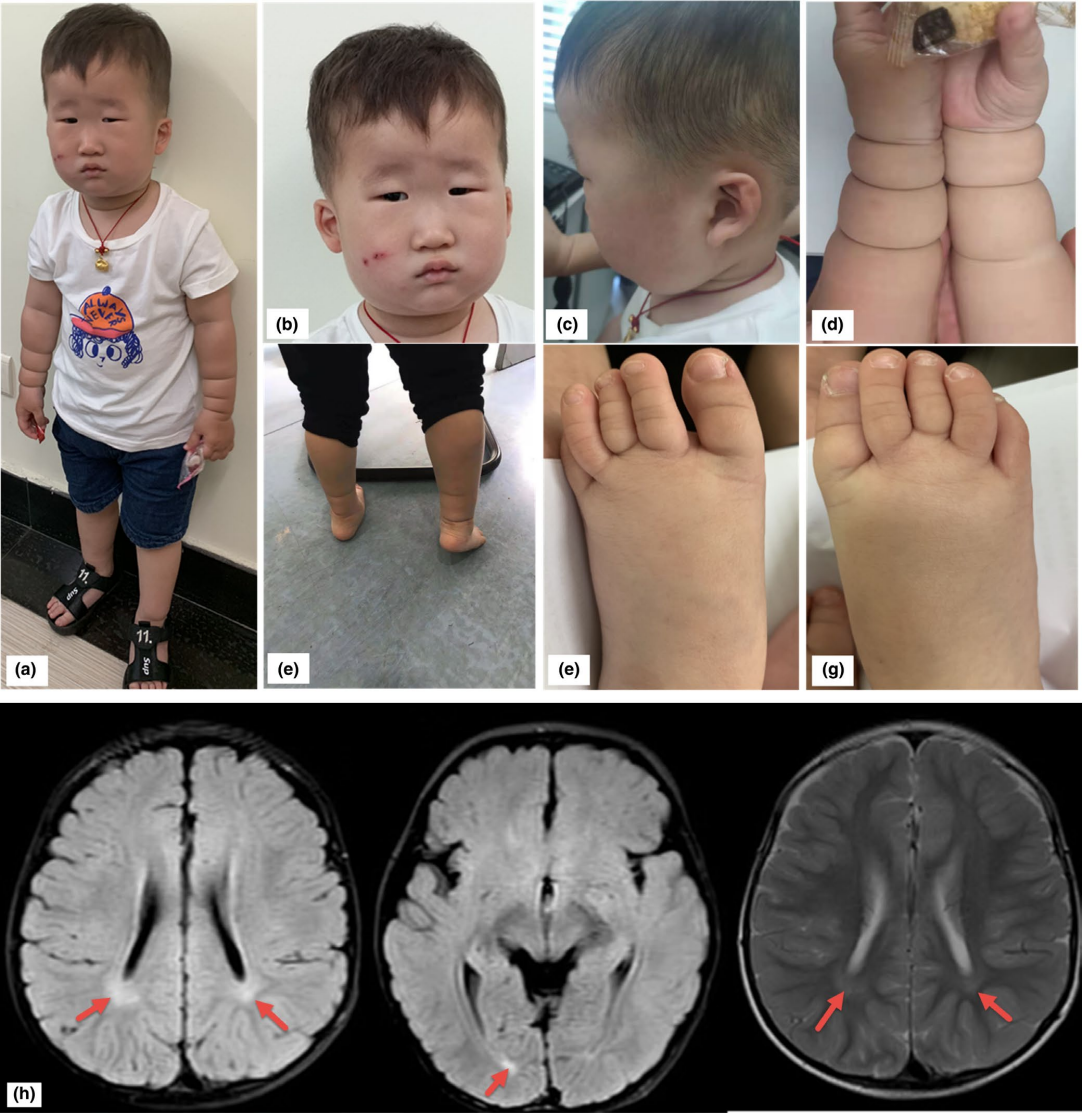
The proband's clinical features at 2 years and 3 months of age. (a) The proband's general appearance. (b) Dysmorphic facial features: macrocephaly, micrognathia, bitemporal narrowing, elongated and flat face, blepharophimosis, broad depressed nasal bridge, epicanthic folds, microphthalmia, microstomia. (c) Low‐set ears with fewer folds. (d and e) Circumferential skin creases on forearms and ankles. (f and g) Apparently partial syndactyly‐like malformation on both feet. X‐ray imaging showing normal. (h) The proband's Magnetic resonance imaging (MRI) demonstrating partial agenesis of white matter of posterior portion

The proband was the only child of nonconsanguineous parents of Chinese Han origin. He was born at 38 weeks of gestation age with no complications. He was referred to the Rehabilitation Department of Shanghai Children's Hospital due to no speech development at 2.3 years of age. He was noticed to have remarkable facial dysmorphic features (Figure 1a–c), presenting elongated and flat face, broad depressed nasal bridge, epicanthic folds, micrognathia, bitemporal narrowing, microphthalmia, microstomia, low‐set ears with simple folds. Symmetric circumferential skin creases on forearms and ankles were noticed (Figure 1d,e). The second to fourth toes of both feet have partial syndactyly‐like malformation while normal foot X‐ray was reported (Figure 1e,f). No cleft palate or high palate was observed. Although his expressive language was absent, his perception language was much better, and he was able to understand and follow his parent's instructions properly.

His growth was on average with a height (91 cm, 0 *SD*) and weight (15 kg, 0–2 *SD*) and an above average head circumference (51 cm, 1–2 *SD*). Brain imaging showed mildly enlarged ventricles and partial agenesis of white matter of the posterior portion. Ultrasound imaging detected unilateral cryptorchidism (Figure 1h). Metabolic work‐up and cardiac and abdominal ultrasound were normal.

## 3 GENETIC ANALYSIS

Blood samples were collected from the proband and both unaffected parents. Genomic DNA was extracted from peripheral blood via standard procedures. Exome capture was carried out using IDTxGen^®^ Exome Research Panel (IDT, USA) and paired‐end sequencing was performed on HiseqX10 (Illumia, USA) to obtain 2 × 75 reads. Sequencing data of all three samples were analyzed to identify sequence variants (single nucleotide variant, SNV; insertion/deletion, Ins/Del) and copy number variants (CNV) using an in‐house pipeline (Fulgent genetics). A phenotype‐driven gene list was created to perform a primary variants interpretation for a more targeted analysis. The clinical significance of variants were interpreted according to the American College of Medical Genetics and Genomics/Association for Molecular Pathology (ACMG/AMP) recommendations (Richards et al., 2015).

## 4 RESULTS

**Figure 2:**
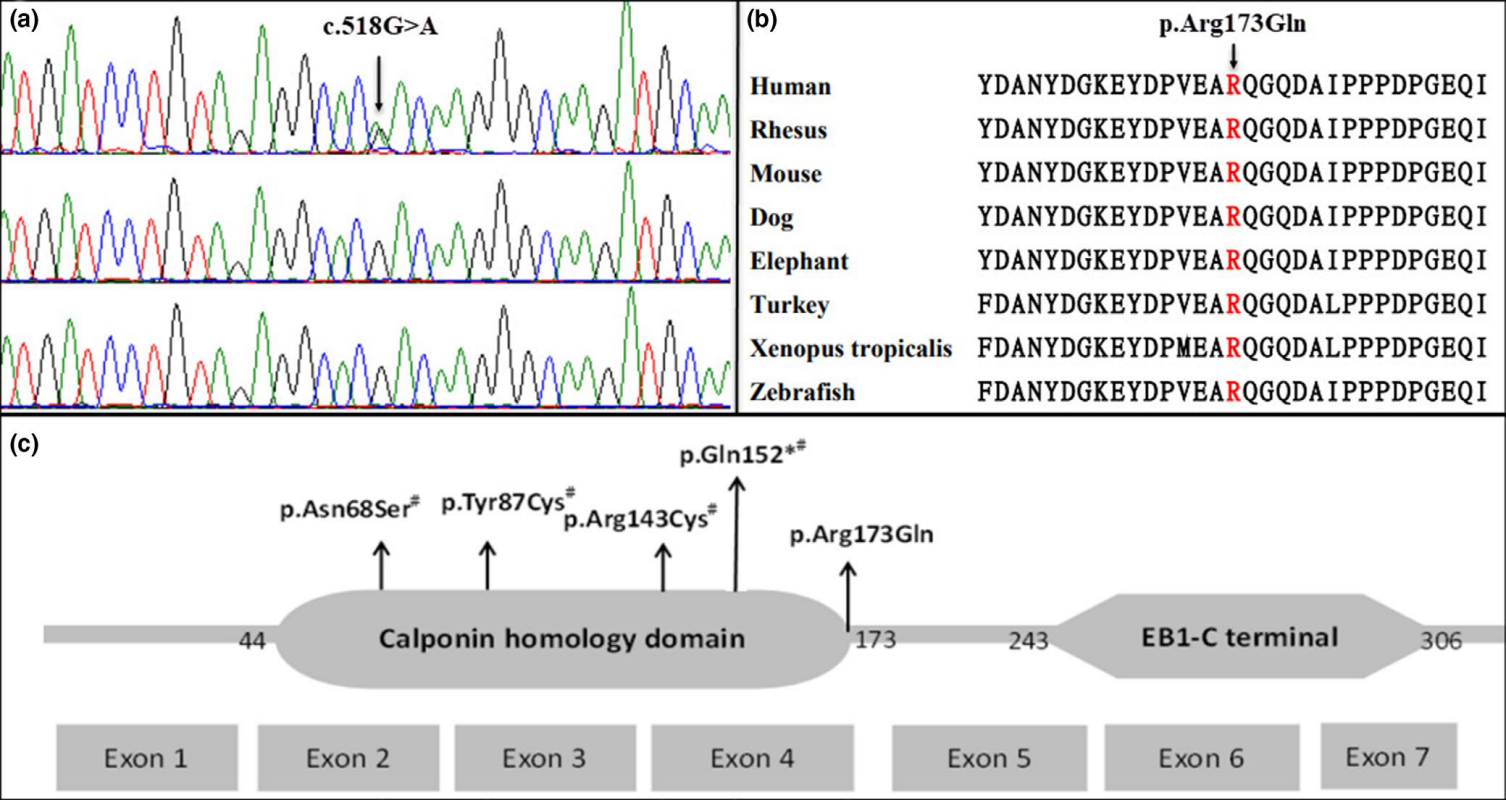
Analysis of *MAPRE2* variants. (a) Chromatogram showing the de novo heterozygous NM_013268.3 (*MAPRE2*):c.518G>A (p.Arg173Gln) variant detected in the proband but not in either parent. Exome sequencing data confirmed biological parental identities. (b) The arginine residue at codon 173 of *MAPRE2* is highly conserved across multiple species. (c) An illustration of the *MAPRE2* gene and functional domains with pathogenic variants identified the present study and previously described patients labeled. All detected *MAPRE2* pathogenic variants reside in the calponin homology domain. ^#^ *MAPRE2* variants previously reported (Isrie et al., 2015)

Written informed consents for the use of photographs and research findings were obtained from the parents. After data analysis, variant filtering, and prioritization, we identified a heterozygous missense variant, NM_014268.3: c.518G>A (p.Arg173Gln). Trio‐based exome analysis showed that the variant was de novo in the proband (Figure 2a). This variant was not present in the Genome Aggregation Database (gnomAD). Analysis of amino acid conservation revealed that the affected amino acid, Arg173, is highly conserved in mammals, including 12/12 primates, and in 38/38 nonmammalian vertebrates (Figure 2b). In silico analysis performed with Alamut Visual suggested potential function effect of the novel missense variant identified in *MAPRE2* is damaging. This residue is located in the C‐terminal of a calponin homology domain of MAPRE2 protein (Figure 2c), where pathogenic variants were enriched. The substitution with Gln173 at this location will probably disrupt its secondary structure, affecting the microtubules binding to this domain. According to the ACMG/AMP guidelines, this variant was considered to be pathogenic based on the evidence of de novo with parental identity confirmed (PS2), absence from large population databases (PM2), located in hot spot functional domain (PM1), multiple lines of computational evidence supporting pathogenicity (PP3) and consistent specific phenotype and inheritance pattern (PP4).

## 5 DISCUSSION

Three missense and one nonsense variants in *MAPRE2* have been hitherto reported in 4 unrelated individuals with CSCSC. Here we report an additional patient with a de novo *MAPRE2* variant that has not been previously reported, which expanded the phenotypic and genetic spectrum of *MAPRE2*‐related CSCSC. To the best of our knowledge, the variant detected in this study is the first reported pathogenic variant in *MAPRE2* identified in a patient with Asian ethnicity. In terms of ClinGen gene curation policy, our findings strengthened the gene–disease association of MAPRE2 and CSCSC2 from “limited” to “moderate”.

MAPRE2 is a microtubule‐associated protein mainly expressed in the central nervous system, including the cerebral cortex, the cerebellum and the spinal cord, and moderately expressed in the skin (GTEx, http://gtexportal.org/home/). It is essential for microtubule reorganization during early stage of apico‐basal epithelial differentiation (Goldspink et al., 2013). Isrie and colleagues found *MAPRE2* mutations had no effect on the secondary or tertiary structure of the protein; interestingly, an enhanced binding of mutant proteins to microtubules was observed in comparison to that of wild‐type control proteins (Isrie et al., 2015). However, the further evidence argued against dominant‐negative effect of the variants, which was considered to cause the disease either through excessive activity or through haploinsufficiency under different paradigms (Isrie et al., 2015). The calponin homology domain of MAPRE2 is a known microtubule‐binding region. Of note, all pathogenic variants detected in CSCSC2 patients reside in this functional domain (Figure 2c), and the one in the present study is located in the last residue of it.

Analysis of the clinical information available for the previously reported patients with *MAPRE2* variants indicated growth parameters, including length and head circumference appear generally below average (0 *SD* to −3 *SD*) postnatally. By contrast, the patient in this study presented with average or mild over postnatal growth for length, weight and head circumference, which differs from previously described clinical features of this disorder. This difference may be due to the different ethnical background. It's not rare that high variable phenotypes were observed between populations with different genetic background for rare diseases even caused by same genes. Notably, the proband in this study also had partial syndactyly‐like malformation instead of cutaneous syndactyly described previously in other patients. Consistently, all five patients, including the present case, were reported to have multiple dysmorphic features including flat face, microphthalmia, microstomia, low‐set and dysmorphic ears, short neck. In terms of the intellectual development, patients with heterozygous variants in *MAPRE2* had moderate to normal intelligence, while homozygous patients presented more severe intellectual disability (Isrie et al., 2015). The present patient had moderate developmental delay with absent expressive language. The protein encoded by *MAPRE2* shares significant homology to the adenomatous polyposis coli protein‐binding EB1 gene family. This protein is a microtubule‐associated protein that is necessary for spindle symmetry during mitosis. It is thought to play a role in the tumorigenesis of colorectal cancers and the proliferative control of normal cells. Whether the patients with *MAPRE2* pathogenic mutations have an increased risk of tumor formation is not well understood yet. A long‐term follow‐up of the patients is necessary.

In conclusion, we identified a previously unreported de novo *MAPRE2* variant in the calponin homology domain in a Chinese patient diagnosed as CSCSC2 with additional clinical features including normal to mild overgrowth, instead of previously reported growth delay, which expanded the clinical and genetic spectrum of MAPRE2‐related CSCSC.

## CONFLICT OF INTEREST

The authors have no conflicts of interests to declare.

## Data Availability

The data used to support the findings of this study are available from the corresponding author upon request.
